# Supplementary material for: Identification of Inhibitors and Drug Targets for Human Adenovirus Infections
Source: Viruses. 2022 May 4;14(5):959. doi: 10.3390/v14050959 (PMC9144521; doi:10.3390/v14050959)
Supplement: Supplementary file 1 [file viruses-14-00959-s001.zip › viruses-1700485-supplementary.pdf]

Literature with reported antiviral activities of the hit compounds identified in this study

Supplemental table S1. Hits of HSP90 inhibitors reported to inhibit virus replication

Target	Class	Hit compound	Reported antiviral activities
PAK		PF-3758309	HIV [1]
HSP90	Resorcinol	STA-9090 (Ganetespib)	EBV [7], HPV [9], RUBV [8],
		AT13387 (Onalespib)	EBV [10]
		HSP990	
		AUY922 (Luminespib)	FMDV [2], HIV [4, 5], KSHV [6], MEV [3], NIV [3], VSV [3]
		VER-49009	
		NMS-E973	
	GM	Geldanamycin	CHIKV [17], EBOV [24], EBV [18], EMCV [11], EV71 [28, 29], HBV [19, 27], HCV [16, 26], HCMV [13, 20], HEV [22], HIV [5], HSV [12], IAV [25], JEV [23], KSHV [30], MeV [3], NIV [3], PRRSV [14], RSV [14], PRV [15], VSV [3,21]
		17-AAG (tanespimycin)	CHIKV [31], EBOV [24], EBV [35], ADV [33], HCMV [20], HBV [39], HCV [32], HIV [4, 5], HSV [34], IAV [25], KSHV [30], MuV [36], PRRSV [14], RSV [14], RUBV [8], RV [37], MERS-CoV [38], SARS-CoV-2 [38, 73], EV71 [28]
		17-DMAG (Alvespimycin)	EBOV [42], EBV [40], FMDV [2], HCV [44], HCMV [43], HIV [5] , KSHV [43], MeV [3], NIV [3], NV [41], VSV [3]
	Indazol-4-one	SNX-2112	CHIKV [17], HSV [45]
		SNX-5422 (PF-04929113)	SARS-CoV-2 [46]
	tropane	XL888	
	triazine	CH5138303	
	Purine	PU-H71(Zelavespib)	KSHV [6]
		BIIB021	KSHV [6]

1. Vargas, B.; Giacobbi, N.S.; Sanyal, A.; Venkatachari, N.J.; Han, F.; Gupta, P.; Sluis-Cremer, N. Inhibitors of Signaling Pathways That Block Reversal of HIV-1 Latency. *Antimicrob Agents Chemother* **2019**, *63*, doi:10.1128/AAC.01744-18.
2. Newman, J.; Asfor, A.S.; Berryman, S.; Jackson, T.; Curry, S.; Tuthill, T.J. The Cellular Chaperone Heat Shock Protein 90 Is Required for Foot-and-Mouth Disease Virus Capsid Precursor Processing and Assembly of Capsid Pentamers. *J Virol* **2018**, *92*, doi:10.1128/JVI.01415-17.
3. Bloyet, L.M.; Welsch, J.; Enchery, F.; Mathieu, C.; de Breyne, S.; Horvat, B.; Grigorov, B.; Gerlier, D. HSP90 Chaperoning in Addition to Phosphoprotein Required for Folding but Not for Supporting Enzymatic Activities of Measles and Nipah Virus L Polymerases. *J Virol* **2016**, *90*, 6642-6656, doi:10.1128/JVI.00602-16.
4. Joshi, P.; Maidji, E.; Stoddart, C.A. Inhibition of heat shock protein 90 prevents HIV rebound. *Journal of Biological Chemistry* **2016**, *291*, 10332-10346.
5. Anderson, I.; Low, J.S.; Weston, S.; Weinberger, M.; Zhyvoloup, A.; Labokha, A.A.; Corazza, G.; Kitson, R.A.; Moody, C.J.; Marcello, A.; et al. Heat shock protein 90 controls HIV-1 reactivation from latency. *Proc Natl Acad Sci U S A* **2014**, *111*, E1528-1537, doi:10.1073/pnas.1320178111.

6. Chen, W.; Sin, S.H.; Wen, K.W.; Damania, B.; Dittmer, D.P. Hsp90 inhibitors are efficacious against Kaposi Sarcoma by enhancing the degradation of the essential viral gene LANA, of the viral co-receptor EphA2 as well as other client proteins. *PLoS Pathog* **2012**, *8*, e1003048, doi:10.1371/journal.ppat.1003048.
7. Shatzer, A.; Ali, M.A.; Chavez, M.; Dowdell, K.; Lee, M.J.; Tomita, Y.; El-Hariry, I.; Trepel, J.B.; Proia, D.A.; Cohen, J.I. Ganetespib, an HSP90 inhibitor, kills Epstein-Barr virus (EBV)-infected B and T cells and reduces the percentage of EBV-infected cells in the blood. *Leuk Lymphoma* **2017**, *58*, 923-931, doi:10.1080/10428194.2016.1213823.
8. Sakata, M.; Katoh, H.; Otsuki, N.; Okamoto, K.; Nakatsu, Y.; Lim, C.K.; Saijo, M.; Takeda, M.; Mori, Y. Heat Shock Protein 90 Ensures the Integrity of Rubella Virus p150 Protein and Supports Viral Replication. *J Virol* **2019**, *93*, doi:10.1128/JVI.01142-19.
9. Patel, K.; Wen, J.; Magliocca, K.; Muller, S.; Liu, Y.; Chen, Z.G.; Saba, N.; Diaz, R. Heat shock protein 90 (HSP90) is overexpressed in p16-negative oropharyngeal squamous cell carcinoma, and its inhibition in vitro potentiates the effects of chemoradiation. *Cancer Chemother Pharmacol* **2014**, *74*, 1015-1022, doi:10.1007/s00280-014-2584-8.
10. Chan, K.C.; Ting, C.M.; Chan, P.S.; Lo, M.C.; Lo, K.W.; Curry, J.E.; Smyth, T.; Lee, A.W.; Ng, W.T.; Tsao, G.S.; et al. A novel Hsp90 inhibitor AT13387 induces senescence in EBV-positive nasopharyngeal carcinoma cells and suppresses tumor formation. *Mol Cancer* **2013**, *12*, 128, doi:10.1186/1476-4598-12-128.
11. Li, Q.; Li, X.; Wu, B.; Niu, Y.; Ma, R.; Xie, J.; Ali, A.; Feng, R. Host protein, HSP90beta, antagonizes IFN-beta signaling pathway and facilitates the proliferation of encephalomyocarditis virus in vitro. *Virus Res* **2021**, *305*, 198547, doi:10.1016/j.virusres.2021.198547.
12. Li, Y.H.; Lu, Q.N.; Wang, H.Q.; Tao, P.Z.; Jiang, J.D. Geldanamycin, a ligand of heat shock protein 90, inhibits herpes simplex virus type 2 replication both in vitro and in vivo. *J Antibiot (Tokyo)* **2012**, *65*, 509-512, doi:10.1038/ja.2012.67.
13. Basha, W.; Kitagawa, R.; Uhara, M.; Imazu, H.; Uechi, K.; Tanaka, J. Geldanamycin, a potent and specific inhibitor of Hsp90, inhibits gene expression and replication of human cytomegalovirus. *Antivir Chem Chemother* **2005**, *16*, 135-146, doi:10.1177/095632020501600206.
14. Gao, J.; Xiao, S.; Liu, X.; Wang, L.; Zhang, X.; Ji, Q.; Wang, Y.; Mo, D.; Chen, Y. Inhibition of HSP90 attenuates porcine reproductive and respiratory syndrome virus production in vitro. *Virol J* **2014**, *11*, 17, doi:10.1186/1743-422X-11-17.
15. Zhang, W.J.; Wang, R.Q.; Li, L.T.; Fu, W.; Chen, H.C.; Liu, Z.F. Hsp90 is involved in pseudorabies virus virion assembly via stabilizing major capsid protein VP5. *Virology* **2021**, *553*, 70-80, doi:10.1016/j.virol.2020.10.013.
16. Okamoto, T.; Nishimura, Y.; Ichimura, T.; Suzuki, K.; Miyamura, T.; Suzuki, T.; Moriishi, K.; Matsuura, Y. Hepatitis C virus RNA replication is regulated by FKBP8 and Hsp90. *EMBO J* **2006**, *25*, 5015-5025, doi:10.1038/sj.emboj.7601367.
17. Rathore, A.P.; Haystead, T.; Das, P.K.; Merits, A.; Ng, M.L.; Vasudevan, S.G. Chikungunya virus nsP3 & nsP4 interacts with HSP-90 to promote virus replication: HSP-90 inhibitors reduce CHIKV infection and inflammation in vivo. *Antiviral Res* **2014**, *103*, 7-16, doi:10.1016/j.antiviral.2013.12.010.
18. Jeon, Y.K.; Park, C.H.; Kim, K.Y.; Li, Y.C.; Kim, J.; Kim, Y.A.; Paik, J.H.; Park, B.K.; Kim, C.W.; Kim, Y.N. The heat-shock protein 90 inhibitor, geldanamycin, induces apoptotic cell death in Epstein-Barr virus-positive NK/T-cell lymphoma by Akt down-regulation. *J Pathol* **2007**, *213*, 170-179, doi:10.1002/path.2219.
19. Bouchard, M.J.; Puro, R.J.; Wang, L.; Schneider, R.J. Activation and inhibition of cellular calcium and tyrosine kinase signaling pathways identify targets of the HBx protein involved in hepatitis B virus replication. *J Virol* **2003**, *77*, 7713-7719, doi:10.1128/jvi.77.14.7713-7719.2003.

20. Evers, D.L.; Chao, C.F.; Zhang, Z.; Huang, E.S. 17-allylamino-17-(demethoxy)geldanamycin (17-AAG) is a potent and effective inhibitor of human cytomegalovirus replication in primary fibroblast cells. *Arch Virol* **2012**, *157*, 1971-1974, doi:10.1007/s00705-012-1379-7.
21. Connor, J.H.; McKenzie, M.O.; Parks, G.D.; Lyles, D.S. Antiviral activity and RNA polymerase degradation following Hsp90 inhibition in a range of negative strand viruses. *Virology* **2007**, *362*, 109-119, doi:10.1016/j.virol.2006.12.026.
22. Zheng, Z.Z.; Miao, J.; Zhao, M.; Tang, M.; Yeo, A.E.; Yu, H.; Zhang, J.; Xia, N.S. Role of heat-shock protein 90 in hepatitis E virus capsid trafficking. *J Gen Virol* **2010**, *91*, 1728-1736, doi:10.1099/vir.0.019323-0.
23. Hung, C.Y.; Tsai, M.C.; Wu, Y.P.; Wang, R.Y.L. Identification of heat-shock protein 90 beta in Japanese encephalitis virus-induced secretion proteins. *J Gen Virol* **2011**, *92*, 2803-2809, doi:10.1099/vir.0.033993-0.
24. Smith, D.R.; McCarthy, S.; Chrovian, A.; Olinger, G.; Stossel, A.; Geisbert, T.W.; Hensley, L.E.; Connor, J.H. Inhibition of heat-shock protein 90 reduces Ebola virus replication. *Antiviral Res* **2010**, *87*, 187-194, doi:10.1016/j.antiviral.2010.04.015.
25. Chase, G.; Deng, T.; Fodor, E.; Leung, B.W.; Mayer, D.; Schwemmle, M.; Brownlee, G. Hsp90 inhibitors reduce influenza virus replication in cell culture. *Virology* **2008**, *377*, 431-439, doi:10.1016/j.virol.2008.04.040.
26. Waxman, L.; Whitney, M.; Pollok, B.A.; Kuo, L.C.; Darke, P.L. Host cell factor requirement for hepatitis C virus enzyme maturation. *Proc Natl Acad Sci U S A* **2001**, *98*, 13931-13935, doi:10.1073/pnas.241510898.
27. Hu, J.; Seeger, C. Hsp90 is required for the activity of a hepatitis B virus reverse transcriptase. *Proc Natl Acad Sci U S A* **1996**, *93*, 1060-1064, doi:10.1073/pnas.93.3.1060.
28. Tsou, Y.L.; Lin, Y.W.; Chang, H.W.; Lin, H.Y.; Shao, H.Y.; Yu, S.L.; Liu, C.C.; Chitra, E.; Sia, C.; Chow, Y.H. Heat shock protein 90: role in enterovirus 71 entry and assembly and potential target for therapy. *PLoS One* **2013**, *8*, e77133, doi:10.1371/journal.pone.0077133.
29. Wang, R.Y.; Kuo, R.L.; Ma, W.C.; Huang, H.I.; Yu, J.S.; Yen, S.M.; Huang, C.R.; Shih, S.R. Heat shock protein-90-beta facilitates enterovirus 71 viral particles assembly. *Virology* **2013**, *443*, 236-247, doi:10.1016/j.virol.2013.05.001.
30. Higashi, C.; Saji, C.; Yamada, K.; Kagawa, H.; Ohga, R.; Taira, T.; Fujimuro, M. The effects of heat shock protein 90 inhibitors on apoptosis and viral replication in primary effusion lymphoma cells. *Biol Pharm Bull* **2012**, *35*, 725-730, doi:10.1248/bpb.35.725.
31. Nayak, T.K.; Mamidi, P.; Kumar, A.; Singh, L.P.; Sahoo, S.S.; Chattopadhyay, S.; Chattopadhyay, S. Regulation of Viral Replication, Apoptosis and Pro-Inflammatory Responses by 17-AAG during Chikungunya Virus Infection in Macrophages. *Viruses* **2017**, *9*, doi:10.3390/v9010003.
32. Ujino, S.; Yamaguchi, S.; Shimotohno, K.; Takaku, H. Combination therapy for hepatitis C virus with heat-shock protein 90 inhibitor 17-AAG and proteasome inhibitor MG132. *Antivir Chem Chemother* **2010**, *20*, 161-167, doi:10.3851/IMP1479.
33. Dalidowska, I.; Gazi, O.; Sulejczak, D.; Przybylski, M.; Bieganowski, P. Heat Shock Protein 90 Chaperones E1A Early Protein of Adenovirus 5 and Is Essential for Replication of the Virus. *Int J Mol Sci* **2021**, *22*, doi:10.3390/ijms22042020.
34. Qin, S.; Hu, X.; Lin, S.; Xiao, J.; Wang, Z.; Jia, J.; Song, X.; Liu, K.; Ren, Z.; Wang, Y. Hsp90 Inhibitors Prevent HSV-1 Replication by Directly Targeting UL42-Hsp90 Complex. *Front Microbiol* **2021**, *12*, 797279, doi:10.3389/fmicb.2021.797279.

35. Murata, T.; Iwata, S.; Siddiquey, M.N.; Kanazawa, T.; Goshima, F.; Kawashima, D.; Kimura, H.; Tsurumi, T. Heat shock protein 90 inhibitors repress latent membrane protein 1 (LMP1) expression and proliferation of Epstein-Barr virus-positive natural killer cell lymphoma. *PLoS One* **2013**, *8*, e63566, doi:10.1371/journal.pone.0063566.
36. Katoh, H.; Kubota, T.; Nakatsu, Y.; Tahara, M.; Kidokoro, M.; Takeda, M. Heat Shock Protein 90 Ensures Efficient Mumps Virus Replication by Assisting with Viral Polymerase Complex Formation. *J Virol* **2017**, *91*, doi:10.1128/JVI.02220-16.
37. Dutta, D.; Bagchi, P.; Chatterjee, A.; Nayak, M.K.; Mukherjee, A.; Chattopadhyay, S.; Nagashima, S.; Kobayashi, N.; Komoto, S.; Taniguchi, K.; et al. The molecular chaperone heat shock protein-90 positively regulates rotavirus infectionx. *Virology* **2009**, *391*, 325-333, doi:10.1016/j.virol.2009.06.044.
38. Li, C.; Chu, H.; Liu, X.; Chiu, M.C.; Zhao, X.; Wang, D.; Wei, Y.; Hou, Y.; Shuai, H.; Cai, J.; et al. Human coronavirus dependency on host heat shock protein 90 reveals an antiviral target. *Emerg Microbes Infect* **2020**, *9*, 2663-2672, doi:10.1080/22221751.2020.1850183.
39. Liu, K.; Qian, L.; Wang, J.; Li, W.; Deng, X.; Chen, X.; Sun, W.; Wei, H.; Qian, X.; Jiang, Y.; et al. Two-dimensional blue native/SDS-PAGE analysis reveals heat shock protein chaperone machinery involved in hepatitis B virus production in HepG2.2.15 cells. *Mol Cell Proteomics* **2009**, *8*, 495-505, doi:10.1074/mcp.M800250-MCP200.
40. Sun, X.; Bristol, J.A.; Iwahori, S.; Hagemeier, S.R.; Meng, Q.; Barlow, E.A.; Fingeroth, J.D.; Tarakanova, V.L.; Kalejta, R.F.; Kenney, S.C. Hsp90 inhibitor 17-DMAG decreases expression of conserved herpesvirus protein kinases and reduces virus production in Epstein-Barr virus-infected cells. *J Virol* **2013**, *87*, 10126-10138, doi:10.1128/JVI.01671-13.
41. Vashist, S.; Urena, L.; Gonzalez-Hernandez, M.B.; Choi, J.; de Rougemont, A.; Rocha-Pereira, J.; Neyts, J.; Hwang, S.; Wobus, C.E.; Goodfellow, I. Molecular chaperone Hsp90 is a therapeutic target for noroviruses. *J Virol* **2015**, *89*, 6352-6363, doi:10.1128/JVI.00315-15.
42. Dowall, S.D.; Bewley, K.; Watson, R.J.; Vasan, S.S.; Ghosh, C.; Konai, M.M.; Gausdal, G.; Lorens, J.B.; Long, J.; Barclay, W.; et al. Antiviral Screening of Multiple Compounds against Ebola Virus. *Viruses* **2016**, *8*, doi:10.3390/v8110277.
43. Wen, K.W.; Damania, B. Hsp90 and Hsp40/Erdj3 are required for the expression and anti-apoptotic function of KSHV K1. *Oncogene* **2010**, *29*, 3532-3544, doi:10.1038/onc.2010.124.
44. Kim, M.G.; Moon, J.S.; Kim, E.J.; Lee, S.H.; Oh, J.W. Destabilization of PDK1 by Hsp90 inactivation suppresses hepatitis C virus replication through inhibition of PRK2-mediated viral RNA polymerase phosphorylation. *Biochem Biophys Res Commun* **2012**, *421*, 112-118, doi:10.1016/j.bbrc.2012.03.126.
45. Xiang, Y.F.; Qian, C.W.; Xing, G.W.; Hao, J.; Xia, M.; Wang, Y.F. Anti-herpes simplex virus efficacies of 2-aminobenzamide derivatives as novel HSP90 inhibitors. *Bioorg Med Chem Lett* **2012**, *22*, 4703-4706, doi:10.1016/j.bmcl.2012.05.079.
46. Goswami, R.; Russell, V.S.; Tu, J.J.; Thomas, C.; Hughes, P.; Kelly, F.; Langel, S.N.; Steppe, J.; Palmer, S.M.; Haystead, T.; et al. Oral Hsp90 inhibitor SNX-5422 attenuates SARS-CoV-2 replication and dampens inflammation in airway cells. *iScience* **2021**, *24*, 103412, doi:10.1016/j.isci.2021.103412.

Supplemental table S2. Hits of PI3K/Akt/mTOR inhibitors reported to inhibit virus replication

Target	Hit compound	Reported antiviral activities
S6 Kinase	H 89 2HCl	
PI3K	VS-5584	
	GNE-317	
Akt	LY3023414(Samotolisib)	
mTOR	AZD8055	HCoV-OC43 [48], HTLV-1 [51], RSV [48]
	PP242(Torkinib)	HIV [47], HCoV-OC43 [48], HTLV-1 [51]
	OSI-027	MERS-CoV [52], SARS-CoV-2 [52]
	WYE-125132	
	WAY-600	
	GDC-0980(Apitolisib)	
	PF-04691502	
	AZD2014(Vistusertib)	SARS-CoV-2 [53,58]
	INK 128(Sapanisertib)	HIV [50]
	Torin 2	RSV [48], HCoV-OC43 [48], HIV [47,57], HCMV [49], HSV [49], SARS-CoV-2 [53,58], IAV [54], ZIKV [55], HCV [56], CHIKV [59]
	Voxtalisib	
	CC-223(Onatasertib)	
	GDC-0349	

47. Besnard, E.; Hakre, S.; Kampmann, M.; Lim, H.W.; Hosmane, N.N.; Martin, A.; Bassik, M.C.; Verschueren, E.; Battivelli, E.; Chan, J.; et al. The mTOR Complex Controls HIV Latency. *Cell Host Microbe* **2016**, *20*, 785-797, doi:10.1016/j.chom.2016.11.001.

48. Huynh, H.; Levitz, R.; Huang, R.; Kahn, J.S. mTOR kinase is a therapeutic target for respiratory syncytial virus and coronaviruses. *Scientific reports* **2021**, *11*, 1-14.

49. Moorman, N.J.; Shenk, T. Rapamycin-resistant mTORC1 kinase activity is required for herpesvirus replication. *J Virol* **2010**, *84*, 5260-5269, doi:10.1128/JVI.02733-09.

50. Heredia, A.; Le, N.; Gartenhaus, R.B.; Sausville, E.; Medina-Moreno, S.; Zapata, J.C.; Davis, C.; Gallo, R.C.; Redfield, R.R. Targeting of mTOR catalytic site inhibits multiple steps of the HIV-1 lifecycle and suppresses HIV-1 viremia in humanized mice. *Proc Natl Acad Sci U S A* **2015**, *112*, 9412-9417, doi:10.1073/pnas.1511144112.

51. Kawata, T.; Tada, K.; Kobayashi, M.; Sakamoto, T.; Takiuchi, Y.; Iwai, F.; Sakurada, M.; Hishizawa, M.; Shirakawa, K.; Shindo, K.; et al. Dual inhibition of the mTORC1 and mTORC2 signaling pathways is a promising therapeutic target for adult T-cell leukemia. *Cancer Sci* **2018**, *109*, 103-111, doi:10.1111/cas.13431.
52. Zhang, X.; Chu, H.; Chik, K.K.; Wen, L.; Shuai, H.; Yang, D.; Wang, Y.; Hou, Y.; Yuen, T.T.; Cai, J.P.; et al. hnRNP C modulates MERS-CoV and SARS-CoV-2 replication by governing the expression of a subset of circRNAs and cognitive mRNAs. *Emerg Microbes Infect* **2022**, *11*, 519-531, doi:10.1080/22221751.2022.2032372.
53. Mullen, P.J.; Garcia, G., Jr.; Purkayastha, A.; Matulionis, N.; Schmid, E.W.; Momcilovic, M.; Sen, C.; Langerman, J.; Ramaiah, A.; Shackelford, D.B.; et al. SARS-CoV-2 infection rewires host cell metabolism and is potentially susceptible to mTORC1 inhibition. *Nat Commun* **2021**, *12*, 1876, doi:10.1038/s41467-021-22166-4.
54. Kuss-Duerkop, S.K.; Wang, J.; Mena, I.; White, K.; Metreveli, G.; Sakthivel, R.; Mata, M.A.; Munoz-Moreno, R.; Chen, X.; Krammer, F.; et al. Influenza virus differentially activates mTORC1 and mTORC2 signaling to maximize late stage replication. *PLoS Pathog* **2017**, *13*, e1006635, doi:10.1371/journal.ppat.1006635.
55. Sahoo, B.R.; Pattnaik, A.; Annamalai, A.S.; Franco, R.; Pattnaik, A.K. Mechanistic Target of Rapamycin Signaling Activation Antagonizes Autophagy To Facilitate Zika Virus Replication. *J Virol* **2020**, *94*, doi:10.1128/JVI.01575-20.
56. Johri, M.K.; Lashkari, H.V.; Gupta, D.; Vedagiri, D.; Harshan, K.H. mTORC1 restricts hepatitis C virus RNA replication through ULK1-mediated suppression of miR-122 and facilitates post-replication events. *J Gen Virol* **2020**, *101*, 86-95, doi:10.1099/jgv.0.001356.
57. Docando, F.; Casado, C.; Pernas, M.; Mota-Biosca, A.; Lopez-Galindez, C.; Olivares, I. Characterizing the antiviral effect of an ATR inhibitor on human immunodeficiency virus type 1 replication. *Arch Virol* **2020**, *165*, 683-690, doi:10.1007/s00705-020-04531-8.
58. Garcia, G., Jr.; Sharma, A.; Ramaiah, A.; Sen, C.; Purkayastha, A.; Kohn, D.B.; Parcells, M.S.; Beck, S.; Kim, H.; Bakowski, M.A.; et al. Antiviral drug screen identifies DNA-damage response inhibitor as potent blocker of SARS-CoV-2 replication. *Cell Rep* **2021**, *35*, 108940, doi:10.1016/j.celrep.2021.108940.
59. Mudaliar, P.; Pradeep, P.; Abraham, R.; Sreekumar, E. Targeting cap-dependent translation to inhibit Chikungunya virus replication: selectivity of p38 MAPK inhibitors to virus-infected cells due to autophagy-mediated down regulation of phospho-ERK. *J Gen Virol* 2021, 102, doi: 10.1099/jgv.0.001629.

Supplemental table S3. Hits of Protein Tyrosine Kinase inhibitors reported to inhibit virus replication

Target	Hit compound	Reported antiviral activities
EGFR	AZD8931 (Sapitinib)	
	OSI-420 (DesMethyl Erlotinib)	HCV [61-63], DENV [61, 62], WNV [61, 62], ZIKV [61,62], HCMV [61], EBOV [62], CHIKV [62], JUNK [62], HIV [62], RSV [62]
	Dacomitinib	
FGFR	AZD4547	
SYK	R406	
	R788 (Fostamatinib)	
VEGFR	Sorafenib Tosylate	HCV [60], HTV [66], IAV [66], IBV [66],MERS-CoV [64], VSV [66]
	Apatinib	LCMV [65], SARS-CoV-2 [66]
	Regorafenib	IAV [66], IBV [66], HTV [66], VSV [66]
PDGFR	Amuvatinib	
SRC	KW-2449	

60. Himmelsbach, K.; Sauter, D.; Baumert, T.F.; Ludwig, L.; Blum, H.E.; Hildt, E. New aspects of an anti-tumour drug: sorafenib efficiently inhibits HCV replication. *Gut* **2009**, *58*, 1644-1653.

61. Weisberg, E.; Parent, A.; Yang, P.L.; Sattler, M.; Liu, Q.; Liu, Q.; Wang, J.; Meng, C.; Buhrlage, S.J.; Gray, N.; et al. Repurposing of Kinase Inhibitors for Treatment of COVID-19. *Pharm Res* **2020**, *37*, 167, doi:10.1007/s11095-020-02851-7.

62. Bekerman, E.; Neveu, G.; Shulla, A.; Brannan, J.; Pu, S.Y.; Wang, S.; Xiao, F.; Barouch-Bentov, R.; Bakken, R.R.; Mateo, R.; et al. Anticancer kinase inhibitors impair intracellular viral trafficking and exert broad-spectrum antiviral effects. *J Clin Invest* **2017**, *127*, 1338-1352, doi:10.1172/JCI89857.

63. Lupberger, J.; Zeisel, M.B.; Xiao, F.; Thumann, C.; Fofana, I.; Zona, L.; Davis, C.; Mee, C.J.; Turek, M.; Gorke, S.; et al. EGFR and EphA2 are host factors for hepatitis C virus entry and possible targets for antiviral therapy. *Nat Med* **2011**, *17*, 589-595, doi:10.1038/nm.2341.

64. Kindrachuk, J.; Ork, B.; Hart, B.J.; Mazur, S.; Holbrook, M.R.; Frieman, M.B.; Traynor, D.; Johnson, R.F.; Dyall, J.; Kuhn, J.H.; et al. Antiviral potential of ERK/MAPK and PI3K/AKT/mTOR signaling modulation for Middle East respiratory syndrome coronavirus infection as identified by temporal kinome analysis. *Antimicrob Agents Chemother* **2015**, *59*, 1088-1099, doi:10.1128/AAC.03659-14.

65. Wan, W.; Zhu, S.; Li, S.; Shang, W.; Zhang, R.; Li, H.; Liu, W.; Xiao, G.; Peng, K.; Zhang, L. High-Throughput Screening of an FDA-Approved Drug Library Identifies Inhibitors against Arenaviruses and SARS-CoV-2. *ACS Infect Dis* **2021**, *7*, 1409-1422, doi:10.1021/acsinfecdis.0c00486.

66. Lesch, M.; Luckner, M.; Meyer, M.; Weege, F.; Gravenstein, I.; Raftery, M.; Sieben, C.; Martin-Sancho, L.; Imai-Matsushima, A.; Welke, R.W.; et al. RNAi-based small molecule repositioning reveals clinically approved urea-based kinase inhibitors as broadly active antivirals. *PLoS Pathog* **2019**, *15*, e1007601, doi:10.1371/journal.ppat.1007601.

Supplemental table S4. Hits of other inhibitors reported to inhibit virus replication

Pathway	Target	Hit compound	Reported antiviral activities
Cell Cycle	Aurora Kinase	ENMD-2076 L-(+)-Tartaric acid	
		AMG-900	
	CDK	BMS-265246	HIV [67]
		ON123300	
Transmembrane Transporters	CRM1	KPT-276	
		KPT-330 (Selinexor)	SARS-CoV-2 [71]
		KPT-335 (Verdinexor)	RSV [68], EBV [69], HCMV [69], KSHV [69], ADV [69], HPV [69], BKV [69], JCV [69], IAV [70, 72], IBV [70], HIV [69]
MAPK	p38 MAPK	Doramapimod	SARS-CoV-2 [73]
		PH-797804	
		Skepinone-L	
	Raf	RAF265	
		TAK-632	
TGF-beta/Smad	PKC	Go6976	IAV [74], HIV [75], HCMV [76]
Metabolism	IMPDH	Mycophenolate Mofetil	HCV [77], PIV [78], ZIKV [79], SARS-CoV-2 [80], HSV [81], MEV [81], DENV [82], CHIKV [83], IAV [84], IBV [84], FMDV [85], JEV [86], BKV [87], HIV [89], EBV [88]
	DHODH	Vidofludimus	SARS-CoV-2 [90], HCMV [90], HIV-1 [90], HCV [90], LCMV [91]

67. Vargas, B.; Giacobbi, N.S.; Sanyal, A.; Venkatachari, N.J.; Han, F.; Gupta, P.; Sluis-Cremer, N. Inhibitors of Signaling Pathways That Block Reversal of HIV-1 Latency. *Antimicrob Agents Chemother* 2019, 63, doi:10.1128/AAC.01744-18.

68. Mathew, C.; Tamir, S.; Tripp, R.A.; Ghildyal, R. Reversible disruption of XPO1-mediated nuclear export inhibits respiratory syncytial virus (RSV) replication. *Sci Rep* 2021, 11, 19223, doi:10.1038/s41598-021-98767-2.

69. Widman, D.G.; Gornisiewicz, S.; Shacham, S.; Tamir, S. In vitro toxicity and efficacy of verdinexor, an exportin 1 inhibitor, on opportunistic viruses affecting immunocompromised individuals. *PLoS One* 2018, 13, e0200043, doi:10.1371/journal.pone.0200043.

70. Perwitasari, O.; Johnson, S.; Yan, X.; Register, E.; Crabtree, J.; Gabbard, J.; Howerth, E.; Shacham, S.; Carlson, R.; Tamir, S.; et al. Antiviral Efficacy of Verdinexor In Vivo in Two Animal Models of Influenza A Virus Infection. *PLoS One* 2016, 11, e0167221, doi:10.1371/journal.pone.0167221.

71. Kashyap, T.; Murray, J.; Walker, C.J.; Chang, H.; Tamir, S.; Hou, B.; Shacham, S.; Kauffman, M.G.; Tripp, R.A.; Landesman, Y. Selinexor, a novel selective inhibitor of nuclear export, reduces SARS-CoV-2 infection and protects the respiratory system in vivo. *Antiviral Res* 2021, 192, 105115, doi:10.1016/j.antiviral.2021.105115.

72. Perwitasari, O.; Johnson, S.; Yan, X.; Howerth, E.; Shacham, S.; Landesman, Y.; Baloglu, E.; McCauley, D.; Tamir, S.; Tompkins, S.M.; et al. Verdinexor, a novel selective inhibitor of nuclear export, reduces influenza a virus replication in vitro and in vivo. *J Virol* 2014, 88, 10228-10243, doi:10.1128/JVI.01774-14.
73. Raymonda, M.H.; Ciesla, J.H.; Monaghan, M.; Leach, J.; Asantewaa, G.; Smorodintsev-Schiller, L.A.; Lutz, M.M.t.; Schafer, X.L.; Takimoto, T.; Dewhurst, S.; et al. Pharmacologic profiling reveals lapatinib as a novel antiviral against SARS-CoV-2 in vitro. *Virology* 2022, 566, 60-68, doi:10.1016/j.virol.2021.11.008.
74. Mahmoudian, S.; Auerochs, S.; Grone, M.; Marschall, M. Influenza A virus proteins PB1 and NS1 are subject to functionally important phosphorylation by protein kinase C. *J Gen Virol* 2009, 90, 1392-1397, doi:10.1099/vir.0.009050-0.
75. Qatsha, K.A.; Rudolph, C.; Marme, D.; Schachtele, C.; May, W.S. Go 6976, a selective inhibitor of protein kinase C, is a potent antagonist of human immunodeficiency virus 1 induction from latent/low-level-producing reservoir cells in vitro. *Proc Natl Acad Sci U S A* 1993, 90, 4674-4678, doi:10.1073/pnas.90.10.4674.
76. Marschall, M.; Stein-Gerlach, M.; Freitag, M.; Kupfer, R.; van den Bogaard, M.; Stamminger, T. Direct targeting of human cytomegalovirus protein kinase pUL97 by kinase inhibitors is a novel principle for antiviral therapy. *J Gen Virol* 2002, 83, 1013-1023, doi:10.1099/0022-1317-83-5-1013.
77. Ye, L.; Li, J.; Zhang, T.; Wang, X.; Wang, Y.; Zhou, Y.; Liu, J.; Parekh, H.K.; Ho, W. Mycophenolate mofetil inhibits hepatitis C virus replication in human hepatic cells. *Virus Res* 2012, 168, 33-40, doi:10.1016/j.virusres.2012.06.009.
78. Uematsu, J.; Sakai-Sugino, K.; Kihira-Nakanishi, S.; Yamamoto, H.; Hirai, K.; Kawano, M.; Nishio, M.; Tsurudome, M.; O'Brien, M.; Komada, H. Inhibitions of human parainfluenza virus type 2 replication by ribavirin and mycophenolate mofetil are restored by guanosine and S-(4-nitrobenzyl)-6-thioinosine. *Drug Discov Ther* 2019, 13, 314-321, doi:10.5582/ddt.2019.01084.
79. Morales Vasquez, D.; Park, J.G.; Avila-Perez, G.; Nogales, A.; de la Torre, J.C.; Almazan, F.; Martinez-Sobrido, L. Identification of Inhibitors of ZIKV Replication. *Viruses* 2020, 12, doi:10.3390/v12091041.
80. Kato, F.; Matsuyama, S.; Kawase, M.; Hishiki, T.; Katoh, H.; Takeda, M. Antiviral activities of mycophenolic acid and IMD-0354 against SARS-CoV-2. *Microbiol Immunol* 2020, 64, 635-639, doi:10.1111/1348-0421.12828.
81. Cline, J.C.; Nelson, J.D.; Gerzon, K.; Williams, R.H.; Delong, D.C. In vitro antiviral activity of mycophenolic acid and its reversal by guanine-type compounds. *Appl Microbiol* 1969, 18, 14-20, doi:10.1128/am.18.1.14-20.1969.
82. Diamond, M.S.; Zachariah, M.; Harris, E. Mycophenolic acid inhibits dengue virus infection by preventing replication of viral RNA. *Virology* 2002, 304, 211-221, doi:10.1006/viro.2002.1685.
83. Khan, M.; Dhanwani, R.; Patro, I.K.; Rao, P.V.; Parida, M.M. Cellular IMPDH enzyme activity is a potential target for the inhibition of Chikungunya virus replication and virus induced apoptosis in cultured mammalian cells. *Antiviral Res* 2011, 89, 1-8, doi:10.1016/j.antiviral.2010.10.009.
84. To, K.K.W.; Mok, K.Y.; Chan, A.S.F.; Cheung, N.N.; Wang, P.; Lui, Y.M.; Chan, J.F.W.; Chen, H.; Chan, K.H.; Kao, R.Y.T.; et al. Mycophenolic acid, an immunomodulator, has potent and broad-spectrum in vitro antiviral activity against pandemic, seasonal and avian influenza viruses affecting humans. *J Gen Virol* 2016, 97, 1807-1817, doi:10.1099/jgv.0.000512.
85. Mei-Jiao, G.; Shi-Fang, L.; Yan-Yan, C.; Jun-Jun, S.; Yue-Feng, S.; Ting-Ting, R.; Yong-Guang, Z.; Hui-Yun, C. Antiviral effects of selected IMPDH and DHODH inhibitors against foot and mouth disease virus. *Biomed Pharmacother* 2019, 118, 109305, doi:10.1016/j.biopha.2019.109305.

86. Sebastian, L.; Madhusudana, S.N.; Ravi, V.; Desai, A. Mycophenolic acid inhibits replication of Japanese encephalitis virus. *Chemotherapy* 2011, 57, 56-61, doi:10.1159/000321483.

87. Acott, P.; O'Regan, P.A.; Crocker, J.F. Suppression of early and chronic BK polyoma virus replication by mycophenolic acid in Vero cells. *Transpl Int* 2009, 22, 225-231, doi:10.1111/j.1432-2277.2008.00766.x.

88. Alfieri, C.; Allison, A.C.; Kieff, E. Effect of mycophenolic acid on Epstein-Barr virus infection of human B lymphocytes. *Antimicrob Agents Chemother* 1994, 38, 126-129, doi:10.1128/AAC.38.1.126.

89. Ichimura, H.; Levy, J.A. Polymerase substrate depletion: a novel strategy for inhibiting the replication of the human immunodeficiency virus. *Virology* 1995, 211, 554-560, doi:10.1006/viro.1995.1437.

90. Hahn, F.; Wangen, C.; Hage, S.; Peter, A.S.; Dobler, G.; Hurst, B.; Julander, J.; Fuchs, J.; Ruzsics, Z.; Uberla, K.; et al. IMU-838, a Developmental DHODH Inhibitor in Phase II for Autoimmune Disease, Shows Anti-SARS-CoV-2 and Broad-Spectrum Antiviral Efficacy In Vitro. *Viruses* 2020, 12, doi:10.3390/v12121394.

91. Kim, Y.J.; Cubitt, B.; Cai, Y.; Kuhn, J.H.; Vitt, D.; Kohlhof, H.; de la Torre, J.C. Novel Dihydroorotate Dehydrogenase Inhibitors with Potent Interferon-Independent Antiviral Activity against Mammarenaviruses In Vitro. *Viruses* 2020, 12, doi:10.3390/v12080821.
